# Cardiovascular risk profile: Cross-sectional analysis of motivational determinants, physical fitness and physical activity

**DOI:** 10.1186/1471-2458-10-592

**Published:** 2010-10-07

**Authors:** Barbara Sassen, Gerjo Kok, Herman Schaalma, Henri Kiers, Luc Vanhees

**Affiliations:** 1 Department of Health and Lifestyle, University of Applied Sciences, Utrecht, the Netherlands; 2 Department of Work and Social Psychology, Maastricht University, Maastricht, the Netherlands; 3 Department of Rehabilitation Sciences, KU Leuven, Leuven, Belgium

## Abstract

**Background:**

Cardiovascular risk factors are associated with physical fitness and, to a lesser extent, physical activity. Lifestyle interventions directed at enhancing physical fitness in order to decrease the risk of cardiovascular diseases should be extended. To enable the development of effective lifestyle interventions for people with cardiovascular risk factors, we investigated motivational, social-cognitive determinants derived from the Theory of Planned Behavior (TPB) and other relevant social psychological theories, next to physical activity and physical fitness.

**Methods:**

In the cross-sectional Utrecht Police Lifestyle Intervention Fitness and Training (UP-LIFT) study, 1298 employees (aged 18 to 62) were asked to complete online questionnaires regarding social-cognitive variables and physical activity. Cardiovascular risk factors and physical fitness (peak VO_2_) were measured.

**Results:**

For people with one or more cardiovascular risk factors (78.7% of the total population), social-cognitive variables accounted for 39% (p < .001) of the variance in the intention to engage in physical activity for 60 minutes every day. Important correlates of intention to engage in physical activity were attitude (beta = .225, p < .001), self-efficacy (beta = .271, p < .001), descriptive norm (beta = .172, p < .001) and barriers (beta = -.169, p < .01). Social-cognitive variables accounted for 52% (p < .001) of the variance in physical active behaviour (being physical active for 60 minutes every day). The intention to engage in physical activity (beta = .469, p < .001) and self-efficacy (beta = .243, p < .001) were, in turn, important correlates of physical active behavior.

In addition to the prediction of intention to engage in physical activity and physical active behavior, we explored the impact of the intensity of physical activity. The intentsity of physical activity was only significantly related to physical active behavior (beta = .253, p < .01, R^2 ^= .06, p < .001). An important goal of our study was to investigate the relationship between physical fitness, the intensity of physical activity and social-cognitive variables. Physical fitness (R^2 ^= .23, p < .001) was positively associated with physical active behavior (beta = .180, p < .01), self-efficacy (beta = .180, p < .01) and the intensity of physical activity (beta = .238, p < .01).

For people with one or more cardiovascular risk factors, 39.9% had positive intentions to engage in physical activity and were also physically active, and 10.5% had a low intentions but were physically active. 37.7% had low intentions and were physically inactive, and about 11.9% had high intentions but were physically inactive.

**Conclusions:**

This study contributes to our ability to optimize cardiovascular risk profiles by demonstrating an important association between physical fitness and social-cognitive variables. Physical fitness can be predicted by physical active behavior as well as by self-efficacy and the intensity of physical activity, and the latter by physical active behavior.

Physical active behavior can be predicted by intention, self-efficacy, descriptive norms and barriers. Intention to engage in physical activity by attitude, self-efficacy, descriptive norms and barriers. An important input for lifestyle changes for people with one or more cardiovascular risk factors was that for ca. 40% of the population the intention to engage in physical activity was in line with their actual physical active behavior.

## Background

In the prevention of cardiovascular disease, the control of cardiovascular risk factors is of upmost importance. Several risk factors are related to lifestyle behavior, such as physical activity patterns [1-3]. Cardiovascular risk factors, namely abdominal obesity, high blood pressure, low HDL-C cholesterol, elevated triglycerides and elevated blood glucose levels, are also interrelated. These risk factors increase the risk for cardiovascular diseases, type 2 diabetes and all-cause mortality [1,3-5]. Research has demonstrated that physical activity and physical fitness have a preventive effect on cardiovascular disease morbidity and mortality [6-10].

Regular physical activity results in increased cardiorespiratory physical fitness. A graded dose-response change in physical fitness was demonstrated with increasing intensities of exercise training or higher levels of physical activity [11-14]. Physical fitness and physical activity are associated with important health benefits for every individual cardiovascular risk factor, having beneficial effect on lipids, blood pressure and glucose metabolism and on weight [15-18]. Research has also shown that physical fitness and physical activity positively influence the group of interrelated risk factors [1,5,19-24].

In a recent study, we found inverse associations between both physical fitness and physical activity with the clustering of cardiovascular risk factors. With regard to physical activity, it appears that particularly (high) intensity physical activity impacts cardiovascular risk factors. Cardiovascular risk factors are strongly associated with physical fitness and, to a lesser extent, with physical activity. Efforts to improve the cardiovascular risk profile and subsequently prevent cardiovascular morbidity and mortality should focus on physical activity which leads to improved physical fitness [25].

In order to enable the development of effective lifestyle interventions that prevent cardiovascular morbidity related to an inactive lifestyle, the social-cognitive determinants of an active lifestyle should be identified [26,27]. In this study, we investigated physical fitness, the intensity of physical activity and the social-cognitive determinants of intentions to engage in an active lifestyle as posited by the Theory of Planned Behavior (TPB) and other relevant social psychological theories [28-35]. According to the TPB, the intention to engage in physical activity predicts future physical active behavior. Intention is the key motivational determinant of behavior. Behavioral intentions, in turn, are determined by three social-cognitive factors, namely attitude, subjective norm and perceived behavioral control or self-efficacy [35]. Attitude refers to the general evaluation of the behavior, and is determined by behavioral beliefs (perceptions regarding the advantages and disadvantages of the behavior) and perceptions regarding the consequences of the behavior. The subjective norm refers to perceived social approval for the behavior. The subjective norm is determined by normative beliefs and by expectations regarding whether important reference individuals or groups will approve of the behavior. In addition to the subjective norm, the descriptive norm, which can viewed as the behavior of others in the social environment, is important [36]. Perceived behavioral control is one's confidence in one's ability to perform a specific behavior. Perceived behavioral control is determined by control beliefs that are based upon perceptions of opportunities, as well as perceived barriers and required resources. An additional and related concept is self-efficacy [35]. People intend to engage in behaviors when the evaluation of the behavior is positive, when social influence is perceived as influential and when the behavior is considered to be under personal control. According to the Theory of Planned Behavior, the influence of general dispositions and sociodemographic factors are mediated by attitude, subjective norms and perceived behavioral control.

In the prevention of cardiovascular risk factors, lifestyle interventions directed at increasing physical activity and hereby enhancing physical fitness may improve the cardiovascular risk profile. A better understanding of the social-cognitive correlates of intention to engage in physical activity on a daily basis and physical active behavior is imperative. Most studies refer to research on social-cognitive determinants without investigating physical activity and physical fitness in a broader scope. We studied social-cognitive determinants of the intention to engage in physical activity and physical active behavior, as well as extensive self-reported physical activity (in intensity and duration) and measured physical fitness (peak VO_2_).

The aims of this study were first to explore the relative importance of social-cognitive variables and a) the intention to engage in physical activity; b) physical active behavior; c) the intensity of physical activity; and d) physical fitness (peak VO_2_). Secondly we investigated the congruence between a high and low intention to engage in physical activity vs. having physical active or inactive behavior.

## Methods

This cross-sectional study was part of the Utrecht Police Intervention Lifestyle Fitness and Training (UP-LIFT) study, which is a voluntary fitness and lifestyle evaluation for police in Utrecht, the Netherlands. The subjects in the present study comprised 1298 people (874 men and 424 women) aged 18 to 62 who visited our research department of Health and Lifestyle between December 2004 and November 2008. All respondents provided written informed consent and approval for the study was obtained from the Ethics Committee at Utrecht University's Medical Centre. We assessed: 1) motivational, social-cognitive determinants, 2) cardiovascular risk factors, 3) physical fitness (peak VO_2_) and 4) physical activity (intensity and duration). Motivational, social-cognitive determinants, cardiovascular risk factors and physical fitness were measured when the respondent came to the laboratory, the questionnaire measuring physical activity (intensity and duration) was filled in before coming to the laboratory.

### Assessment of motivational social-cognitive determinants

For the assessment of motivational, social-cognitive determinants, participants were asked to complete an online secure website questionnaire. The questionnaire content was derived from a literature study and in-depth interviews on physical activity conducted with people who had cardiovascular risk factors. There are no valid questionnaires for measuring social-cognitive determinants, but these are valid procedures. The construction of the questionnaire is, according to the TPB, specific to the definition of the behavior and the specification of the research population [30,37]. The following motivational, social-cognitive determinants were included in the questionnaire.

*Attitude *was assessed with two items: 'In my view being physically active for 60 minutes every day is very good-very bad' and 'In my view being physically active for 60 minutes every day is very pleasant to do/very unpleasant to do' (Cronbach's α = .73). *Beliefs *were measured by eight items. An example item is: 'Being physically active for 60 minutes every day is very healthy/very unhealthy' (Cronbach's α = .31). *Subjective norm *was measured by two items: 'Most people/most colleagues who are important to me think I should be physically active.' Answer options ranged from 'no, certainly not' to 'yes, certainly' (Cronbach's α = .74). *Descriptive norm *was indexed by one item: 'Are those people who are important to you physically active?'. *Perceived behavioral control *was measured by five items on being physically active even when busy, in pain, etc. (Cronbach's α = .82). S*elf-efficacy *was indexed with three questions referring to one's capability, trust in one's capability and experienced difficulty to be physically active every day (Cronbach's α = .81). *Intention *was measured with two items: 'Do you intend to be physically active tomorrow and the day after tomorrow?' and 'Do you surely intend to be physically active tomorrow and the day after tomorrow?' (Cronbach's α = .93). Intention was also dichotomized in a high and low intention group by categorizing 'definitely yes' as high intention and all other answers as low intention. *Behavior *was measured by averaging two questions: 'Are you physically active for at least 60 minutes a day?' and 'Do you think you engage in adequate physical activity?' Behavior was also dichotomized in active and inactive lifestyle behavior by splitting the averaged item. Those scores above 4.5 were considered active. *Barriers *were measured with five questions about being 'no, certainly not/yes, certainly' physically active for 60 minutes every day even when one is tired, busy, in pain, etc. Responses were provided on a seven-point scale (1 = definitely not, 7 = definitely yes). An inverse scale was used for barriers. A determinant score was calculated by averaging the total score and dividing that average by the number of items.

### Assessment of cardiovascular risk factors

Next to the assessment of social-cognitive determinants we measured cardiovascular risk factors. Respondents were classified without or as having one, two or three or more cardiovascular risk factors. In this study, the group at risk for cardiovascular morbidity was having at least one cardiovascular risk factor. The cardiovascular risk factors are, based on the extended ATP-III criteria put forth by the National Cholesterol Education Program Adult Treatment Panel III definition: 1) abdominal obesity (men: waist circumference > 102 cm; women: > 88 cm); 2) high blood pressure (systolic ≥ 130 mm Hg or diastolic ≥ 90 mmHg) or being on antihypertensive medication; 3) low HDL-C cholesterol (high-density lipoprotein, men < 1.03 mmol/L, women < 1.30 mmol/L) or being on medication for HDL-C; 4) elevated triglycerides (≥ 1.70 mmol/L) or being on medication for elevated triglycerides; and 5) high blood glucose (≥ 6.1 mmol/L) or being on medication for elevated glucose definition [1,4].

### Assessment of physical fitness and physical activity

For the assessment of physical fitness, peak oxygen uptake (peak VO_2_) as the gold standard for exercise capacity, was measured [14]. The individual's maximum cardiorespiratory fitness level was tested using a bicycle ergometer (Siemens-Elema 380B; Ergonomics 800S^®, ^Ergonomics, Bitz, Germany) in a laboratory with a stabilized room temperature. The initial workload of 20 Watts was increased every minute by 20 Watts until volitional exhaustion was reached. During the test, a 12-lead electrocardiogram and respiratory data through breath-by-breath analysis (Oxyxon Pro^®^, Jaeger, Mijnhardt) were continuously measured. Heart rate (HR) was calculated from the electrocardiogram. The gas analyzers and the flow meters were calibrated before each test according to the manufacturer's instructions and oxygen uptake (VO_2_) was determined from the continuous measurement of oxygen concentration in the inspired and expired air.

Next to social-cognitive determinants, cardiovascular risk factors and physical fitness, we assessed physical activity. Physical activity was assessed with the Short Questionnaire to Assess Health Enhancing Physical Activity (SQUASH), of which the reproducibility and validity has been tested and proved to be fairly reproducible and reasonable valid. The overall reproducibility was r = 0.58 (95% CI 0.36-0.74) and the validity when compared to the Computer Science and Applications activity monitor was r = 0.45 (95% CI 0.17-0.66 [38]. Data were collected on a secure website. Participants were asked to report average time (days per week, hours per day, minutes per day) and type of physical activity. For the purposes of our study, we selected walking, cycling and sports activities. We calculated physical activity in intensity and duration. The average intensity of physical activity was expressed as the intensity of physical activity, for this physical activities were given a metabolic equivalent value (METS), ranging from 1 to 12 METS [39]. The time spent on physical activity was expressed as physical activity duration in hours per week. For details see 25.

### Statistical analysis

One-way analyses of variance (ANOVA) and Chi-square analyses were conducted for people without, with one, with two or with three or more cardiovascular risk factors. Subsequently, a correlation matrix of all the social-cognitive variables as well as physical fitness and physical activity was generated for people with one or more cardiovascular risk factors. To explore the congruence between having a high versus low intention to engage in physical activity and physical active versus physical inactive behavior, Chi-square analyses were conducted. Hierarchical regression analyses were applied to model intention to engage in physical activity, physical active behavior, the intensity of physical activity and physical fitness (peak VO_2_). We analyzed the association for people with one or more cardiovascular risk factors between: 1) the intention to engage in physical activity and social-cognitive variables; 2) physical active behavior, intention and social-cognitive variables; 3) the intensity of physical activity, physical active behavior, intention and social-cognitive variables; and 4) physical fitness, the intensity of physical activity, physical active behavior, intention and social-cognitive variables. Multicollinearity between perceived behavioral control and self-efficacy and between physical activity and physical fitness variables was explored and not found. Significance was set at p < .05 and at p < .01 for the hierarchical regression analysis. Statistical analyses were conducted with SPSS version 15.0 (SPSS inc. 2006).

## Results

Table 1 presents descriptive statistics for the cardiovascular risk factor groups, people having resp. zero, one, two or three or more cardiovascular risk factors. Four out of every five participants were classified as having one or more cardiovascular risk factors, with men being more likely to have risk factors than women. Age was positively associated with the number of cardiovascular risk factors. Of the total sample, 18.6% had three or more cardiovascular risk factors. Cardiovascular risk factor groups differed significantly on behavior and social-cognitive correlates, but not on the intention to engage in physical activity. ANOVAs revealed differences for attitude, beliefs, subjective norms and self-efficacy between the different risk factors groups. Cardiovascular risk factor groups differed significantly on physical fitness (peak VO_2_) and intensity of physical activity, but not on duration of physical activity. Both physical fitness and physical activity intensity being negatively associated with the number of cardiovascular risk factors.

**Table 1:** People having 0, 1, 2 or ≥ 3 cardiovascular risk factors and social-cognitive determinants, physical fitness, physical activity intensity and physical activity duration, total population

N = 1298	0 risk factors	1 risk factor	2 risk factors	≥3 risk factors	p
	276 (21.3)	475 (36.6)	305 (23.5)	242 (18.6)	
Male	138 (15.8)	318 (36.4)	221 (25.3)	197 (22.5)	< .001
Female	138 (32.6)	157 (37.0)	84 (19.8)	45 (10.6)	
Age (years)	32.8 ± 9.3^bcd^	37.0 ± 11.1^acd^	39.7 ± 10.8^abd^	44.8 ± 9.4^abc^	< .001
Social-cognitieve determinants:					
Behavior	4.33 ± 0.98^d^	4.31 ± 1.01^d^	4.14 ± 1.05^d^	3.83 ± 1.09^abc^	< .001
Intention	6.30 ± 1.07	6.18 ± 1.14	6.14 ± 1.19	6.05 ± 1.18	ns
Attitude	6.35 ± 0.61^d^	6.25 ± 0.63^d^	6.25 ± 0.67	6.11 ± 0.75^ab^	< .01
Beliefs	4.60 ± 0.52	4.56 ± 0.49^d^	4.62 ± 0.53	4.68 ± 0.56^b^	< .05
Subjective norms	4.83 ± 1.21^cd^	4.90 ± 1.25^cd^	5.13 ± 1.22^ab^	5.21 ± 1.11^ab^	< .001
Perceived control	4.96 ± 0.98	5.07 ± 1.02	4.98 ± 1.02	4.88 ± 1.07	ns
Self-efficacy	5.99 ± 0.94^d^	5.96 ± 0.95^d^	5.80 ± 1.10	5.60 ± 1.16^ab^	< .001
Descriptive norms	3.15 ± 1.09	3.05 ± 1.08	3.10 ± 1.03	3.06 ± 1.13	ns
Barriers	3.09 ± 0.98	3.13 ± 1.05	3.12 ± 1.01	3.24 ± 1.04	ns
					
Physical fitness	38.4 ± 7.28^cd^	37.0 ± 7.78^cd^	34.4 ± 8.12^abd^	31.0 ± 7.49^abc^	< .001
Physical activity intensity	4.95 ± 1.33^cd^	4.86 ± 1.42^d^	4.64 ± 1.53 ^a^	4.33 ± 1.47^ab^	< .001
Physical activity duration	9.66 ± 8.90	9.54 ± 8.56	9.34 ± 9.02	8.07 ± 8.39	ns

Values are numbers (and percentages) or mean ± SD;

Social-cognitive determinants range 1-7;

Abbreviations: P, p-value; a = different from 0 risk factor group, b = different from 1 risk factor group, c = different from the 2 risk factor group, d = different from the ≥3 risk factor group.

The correlation matrix (Table 2) displays positive correlations for people with one or more cardiovascular risk factors between physical active behavior and the key motivational determinant of behavior, namely the intention to engage in physical activity for 60 minutes every day (r = .66). Physical active behavior was found to be positively related to self-efficacy (r = .57), and perceived behavioral control (r = .41) and negatively related to barriers (r = -.43). Associations were also found between physical active behavior and physical fitness (r = .38). The correlation between physical active behavior and physical activity intensity was lower (r = .25). Physical fitness was positively correlated with self-efficacy (r = .37).

**Table 2:** Correlations between social-cognitive determinants, physical fitness and physical activity for people with one or more cardiovascular risk factors

N = 989	Beh	Int	Att	SN	PC	DN	SE	Bel	Barr	PF	PA int	PA dur
Behavior	Beh											
Intention	.66***	Int										
Attitude	.40***	.47***	Att									
Subjective norms	.20***	.32***	.31***	SN								
Perceived control	.41***	.39***	.41***	.24***	PC							
Descriptive norms	.37***	.37***	.27***	.53***	.26***	DN						
Self-efficacy	.57***	.52***	.51***	.32***	.53***	.35***	DSE					
Beliefs	-.07*	ns	.09**	.47***	-.09**	ns	-.12***	Bel				
Barriers	-.43***	-.43***	-.39***	-.27***	-.78***	-.26***	-.48***	ns	Barr			
Physical fitness	.38***	.18***	.17***	ns	.23***	ns	.37***	-.23***	-.24***	PF		
												
Physical activity intensity	.25***	.10**	ns	-.09	ns	ns	.10**	ns	-.073*	.31***	PA-int	
												
Physical activity duration	.44***	.31***	.21***	.11**	.25***	.15***	.28***	ns	-.28***	.23***	ns	PA dur
												
Mean	4.18	6.17	6.24	4.99	4.98	3.08	5.86	4.60	3.16	35.56	4.93	12.52
SD	1.04	1.14	0.66	1.21	1.02	1.08	1.03	0.52	1.03	8.12	1.44	11.34
Range	1-6	1-7	1-7	1-7	1-7	1-7	1-7	1-7	1-7	12-61	0-215	0-215

Values represent correlation coefficients;

P-value;*** < 0.001, ** < 0.01, * < 0.05.

According to the Theory of Planned Behavior, intention is determined by attitude, subjective norm and perceived behavioral control. Positive correlations were found between the intention to engage in physical activity for 60 minutes every day and attitude (r = .47), subjective norm (r = .32), and perceived behavioral control (r = -.39).

### Congruence between intention to engage in physical activity and physical active behavior

Analyses were undertaken to explore sensitivity and specificity for physical active behavior (Table 3). Do people with positive intentions to engage in physical activity for 60 minutes every day actually show physical active behavior? Among people with one or more cardiovascular risk factors, 50.4% reported having positive intentions to engage in physical activity and 51.8% reported physical active behavior. Also, 39.9% of the participants had positive intentions to engage in physical activity and had physical active behavior, while 11.9% had a positive intention but had physical *in*active behavior. A large group (37.7%) scored low on intention to engage in physical activity and had *in*active behavior, while 10.5% scored low on intention but reported physical active behavior. We found that inactive behavior increased as the number of cardiovascular risk factors increased, and that the number of participants with low intentions increased as the number of cardiovascular risk factors increased.

**Table 3:** High and low intention and active and inactive behavior for people with one or more cardiovascular risk factors

N = 989	Behavior		
	**Active**	**Inactive**	**Total**

Intention high	394 (77.0)	104 (21.8)	498 (50.4)
	*39.9*	*11.9*	
Intention low	118 (23.0)	373 (78.2)	491 (49.6)
	*10.5*	*37.7*	
Total	512 (51.8)	477 (48.2)	

Chi-square analysis, p < 0.05;

Values are numbers and (%);

Cursive, percentage of the total group.

### Prediction of intention, physical active behavior, physical activity intensity and physical fitness

We employed (hierarchical) regression analyses to identify correlates of the intention to engage in physical activity, of physical active behavior, of physical activity intensity and of physical fitness for people with one or more cardiovascular risk factors (Table 4). Social-cognitive variables accounted for 39%of the variance (p < .001) in intention to engage in physical activity. Salient variables were attitude (β = .225, p < .001), self-efficacy (β = .271, p < .001), descriptive norm (β = .172, p < .001) and barriers (β = -.169, p < .01). With respect to physical active behavior, 52% of the variance (p < .001) was explained by intention and social-cognitive variables, with intention (β = .469, p < .001) and self-efficacy (β = .243, p < .001) being the strongest predictors

**Table 4:** Prediction of intention, physical active behavior, physical activity intensity and physical fitness for people with one or more cardiovascular risk factors

N = 989	Intention		Physical active behavior		Physical activity intensity		Physical fitness	
	**r**	**β**	**r**	**β**	**r**	**β**	**r**	**β**
Physical activityintensity							.31**	.238**
Behavior					.25**	.253**	.38**	.180**
								
Intention			.66**	.469**	.10*	-	.18**	-
								
Attitude	.47**	.225**	.40**	-	ns	-	.17**	-
Subjective norms	.32**	-	.20**	-	ns	-	ns	-
Perceived control	.39**	-	.41**	-	ns	-	.23**	-
Self-efficacy	.52**	.271**	.57**	.243**	.10**	-	.37**	.180**
Descriptive norms	.37**	.172**	.37**	.084*	ns	-	ns	-
Barriers	-.43**	-.169*	-.43**	-.095**	ns	-	-.24**	-

R^2^		.39**		.52**		.06*		.23**

P-value;** < 0.001, * < 0.01.

In addition to the predicting of intention to engage in physical activity and physical active behavior, we explored the intensity of physical activity. Only 6% of the variance (p < .001) in physical activity intensity was explained by social cognitive variables with physical active behavior (β = .253, p < .01) as significant predictor. An important goal of our research was to investigate the relationship between physical fitness, on the one hand, and the intensity of physical activity and the social-cognitive variables, on the other. In this regression analysis, 23% of the variance (p < .001) in physical fitness was explainedby physical active behavior (β = .180, p < .01), self-efficacy (β = .180, p < .01) and physical activity intensity (β = .238, p < .01).

## Discussion

In order to optimize the cardiovascular risk profile of people with one or more cardiovascular risk factors, this study explored physical fitness and the intensity of physical activity in relation to social-cognitive variables derived from the Theory of Planned Behavior and other relevant social psychological theories. We determined that 23% of the variance in physical fitness could be predicted by physical active behavior, self-efficacy and the intensity of physical activity. These three variables are therefore likely to be important objectives for interventions directed at deceasing cardiovascular morbidity and mortality. The findings also suggest that, in order to optimize the preventive effect of physical fitness, efforts to motivate behavior change by strengthening behavioral intention, strengthen self-efficacy and then later adding intensity to physical activity are worthwhile.

With respect to the mean intensity of physical activity, research has demonstrated that the volume of (high) intensity and, most importantly, physical fitness have an important effect on reducing cardiovascular risk factors [25]. In this study, we showed that the intensity of physical activity is an important predictor of physical fitness and that the intensity of physical activity was, in turn, predicted by physical active behavior. Physical active behavior only accounted for 6% of the variance in the intensity of physical activity.

In addition to physical fitness and physical activity intensity, we explored social-cognitive variables, which are prerequisite inputs for the development of effective lifestyle interventions to prevent cardiovascular risk [27]. We showed that physical active behavior predicted physical fitness and physical activity intensity. We also found that social-cognitive variables are useful predictors of physical active behavior and of the intention to engage in physical activity for 60 minutes every day. Social-cognitive variables accounted for 52% of the variance in physical active behavior and 39% of the variance in intention to engage in physical activity. A meta-analytic review showed that social-cognitive variables accounted for 27% of the variance in behavior and 39% of the variance in intention [40]. Previous studies have shown regression values of 36% for behavior and 42% to 66% for intention [30,41]. In our study, the amount of variance accounted for in behavior was not smaller than the amount of variance accounted for in intention, possible because behavior was better predicted by perceived control and self-efficacy than intention, indicating that participants were accurate in estimating their actual control.

Physical active behavior was largely associated with the intention to engage in physical activity for 60 minutes every day. Self-efficacy, descriptive norms and barriers were also relevant predictors. This corresponds with previous studies in which behavior was found to be largely predicted by intention [32,42]. In our study, self-efficacy was found to be an important predictor of behavior and this is congruent with other research [34]. The claim that perceived behavioral control does not strongly predict behavior once intention is included was supported by our study, although we know self-efficacy and perceived behavioral control has the same underlying concept [32,37].

Four social-cognitive variables explained a significant proportion of the variance in physical active behavior. The results suggest that people with positive intentions and high levels of self-efficacy are more likely to engage in physical active behavior. They also suggest investing in efforts that develop the skills necessary to overcome barriers. According to the Theory of Planned Behavior, intention to engage in physical activity predicts future physical active behavior. Our study confirmed this.

Also, our results indicated that, among people with one or more cardiovascular risk factors, intention to engage in physical activity for 60 minutes every day was strongly associated with attitude and self-efficacy and, to a lesser extent, with descriptive norms and barriers. That attitude and self-efficacy are significant contributors to intention to engage in physical activity is congruent with other research [30,40,41,43]. Our findings also revealed that people with more positive attitudes and higher self-efficacy are more likely to have positive intentions about physical activity. Similar to other studies, our study found subjective norms to be weak predictors of exercise intention [30,31,41]. In our study, the variables attitude and self-efficacy accounted for a large part of the variance in intention. People with one or more cardiovascular risk factors perceived more advantages than disadvantages to engaging in regular physical activity. Interventions should therefore endeavour to highlight and clarify the advantages of engaging in physical activity while providing solutions for the disadvantages. Also, self-efficacy was associated with intention thus suggesting that physical activity is perceived as relatively easy to adopt and that interventions should gradually add intensity to physical activity as physical activity levels improve. Interventions should also support the development of control over physical activity. Subjective norm appeared to be less important. The influence of significant others was found to impact the intention to engage in physical activity for 60 minutes every day. Interventions should therefore be directed at both learning skills necessary to handle negative social influence and strengthening positive social support.

According to a meta-analytic review [40], social-cognitive variables predict self-reported behavior better than observed behavior. In our study, social-cognitive variables were found to be good predicators of intention, behavior and physical fitness. They were less adept at predicting physical activity intensity.

In addition to exploring the contribution of social-cognitive variables to intention and physical active behavior, we explored the congruence between high and low intention vs. having physical active or inactive behaviour [44]. Do people with positive intentions actually engage in physical active behaviour? And, more importantly, how many people have positive intentions but are inactive? Among those with one or more cardiovascular risk factors, about 40% had positive intentions and were physically active, about 40% had low intentions and were *in*active, about 10% had high intentions but were inactive and about 10% had a low intentions but were active. This incongruence between high and low intentions and reporting physically active versus inactive behavior is an important input for lifestyle changes. The social-cognitive determinants of intentions and behaviors may be changed in a more positive direction by planned interventions, deciding which determinants need to be changed, which need to be reinforced, and which need to be introduced [26,27].

The present study had several strengths including its sample size, the inclusion of social-cognitive variables and the simultaneous assessment of physical fitness and physical activity. A limitation is the cross-sectional design of the study and that the study was carried out in a police department.

## Conclusions

This study contributes to our ability to optimize cardiovascular risk profiles by demonstrating an important association between physical fitness and social-cognitive variables. Physical fitness can be predicted by physical active behavior as well as by self-efficacy and the intensity of physical activity, and the latter by physical active behavior.

Physical active behavior can be predicted by intention, self-efficacy, descriptive norms and barriers. Intention to engage in physical activity can be predicted by attitude, self-efficacy, descriptive norms and barriers. An important input for lifestyle changes for people with 1 or more cardiovascular risk factors was that for ca. 40% of the population the intention to engage in physical activity was in line with their actual physical active behavior.

## Competing interests

The authors declare that they have no competing interests.

## Authors' contributions

BS analyzed the data and wrote the manuscript. BS, GK and HS were contributed to the study design and the interpretation of the data. HK prepared the database before data analysis. LV conceived the UP-LIFT study and was responsible for the study design. All authors approved the final manuscript, except HS.

## Pre-publication history

The pre-publication history for this paper can be accessed here:

http://www.biomedcentral.com/1471-2458/10/592/prepub
